# Obstructive shock and cardiac arrest due to diaphragmatic hernia after esophageal surgery: a case report

**DOI:** 10.1186/s40792-024-02071-w

**Published:** 2024-11-19

**Authors:** Kensuke Minami, Rie Nakatsuka, Satoshi Nagaoka, Masaki Hirota, Takashi Matsumoto, Takashi Kusu, Tatsushi Shingai, Yoichi Makari, Satoshi Oshima

**Affiliations:** Department of Surgery, Kinki Central Hospital, 3-1 Kurumazuka, Itami, Hyogo 664-8533 Japan

**Keywords:** Obstructive shock, Postoperative diaphragmatic hernia, Esophageal surgery

## Abstract

**Background:**

We report the exceedingly rare case of diaphragmatic hernia after esophageal surgery resulting in obstructive shock and cardiac arrest.

**Case presentation:**

An 82-year-old man, who had undergone a robotic-assisted thoracoscopic esophagectomy and gastric tube reconstruction via a subcutaneously route with three-field lymphadenectomy for esophagogastric junction cancer at another hospital 3 months prior, complaining of persistent epigastric pain and nausea. Computed tomography revealed that the proximal jejunum had herniated through the esophageal hiatus into the left thoracic cavity, with dilation of the subcutaneous gastric tube and duodenum. He was urgently admitted, and a nasogastric tube was inserted. His respiratory and circulatory parameters were normal upon admission, however, nine hours after admission, there was a rapid increase in oxygen demand, and he subsequently developed shock. His blood pressure was 106/65 mmHg, pulse rate of 150bpm, respiratory rate of 30/min with an O2 saturation of 97% on High-flow nasal cannula FiO2:0.4, cyanosis and peripheral coldness appeared. Chest X-ray showed a severe mediastinal shift to the right, suggesting obstructive shock due to intestinal hernia into the thoracic cavity. Emergency surgery was planned, but shortly after endotracheal intubation, the patient experienced cardiac arrest. It was found that approximately 220 cm of small intestine had herniated into the thoracic cavity through the esophageal hiatus, and it was being strangulated by the diaphragmatic crura. A portion of the diaphragmatic crura was incised to manually reduce the herniated small intestine back into the abdominal cavity. The strangulated intestine was congested, but improvement in coloration was observed and it had not become necrotic. The procedure finished with closure of the esophageal hiatus. Intensive care was continued, but he died on postoperative day 29 because of complications including perforation of the descending colon and aspiration pneumonia.

**Conclusion:**

Rapid progression of small intestine hernia into the thoracic cavity, leading to obstructive shock, was suspected. While this case was rare, early recognition of the condition and prompt reduction could have potentially led to life-saving outcomes.

## Introduction

Obstructive shock is a condition where an acute obstruction of blood flow in central vessels for some reason leads to a reduction in the amount of blood that the heart can pump out, resulting in the inability to maintain vital organ blood flow. Representative conditions of obstructive shock include pulmonary embolism, cardiac tamponade, and tension pneumothorax. We report a case of obstructive shock caused by herniation of intestinal loops into the thoracic cavity after esophageal surgery.

Diaphragmatic hernia after esophageal surgery is relatively rare, with an incidence rate from 0.4 to 4% [1–4]. It is known to be associated with severe respiratory impairment due to compression of the lungs and life-threatening complications [1] such as intestinal necrosis and perforation due to ischemia [3, 5]. Fatal cases of intestinal strangulation have also been reported. However, cases resulting in obstructive shock due to herniated small intestine are extremely rare.

## Case

**Fig. 1:**
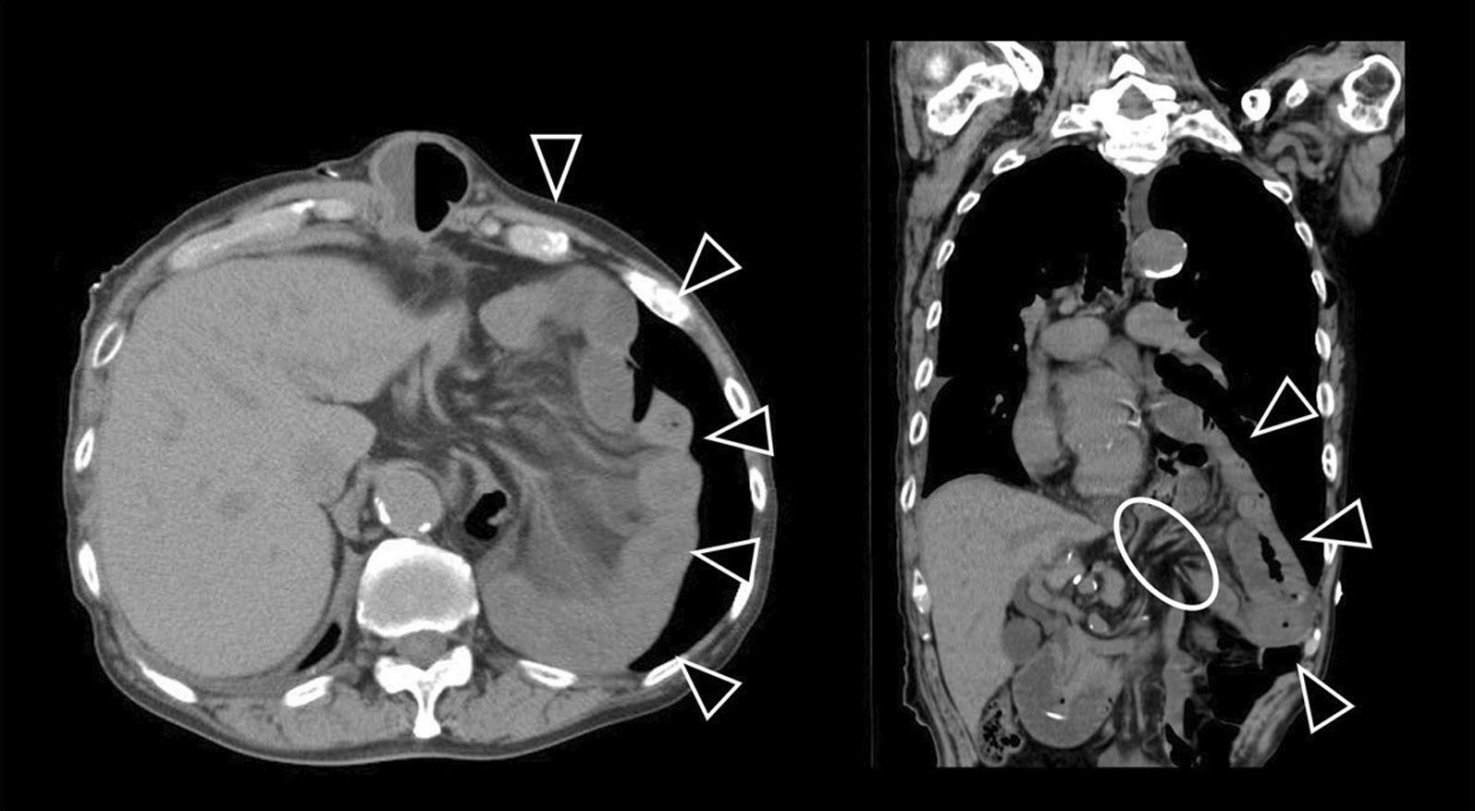
The proximal jejunum had herniated through the esophageal hiatus into the left thoracic cavity

**Fig. 2:**
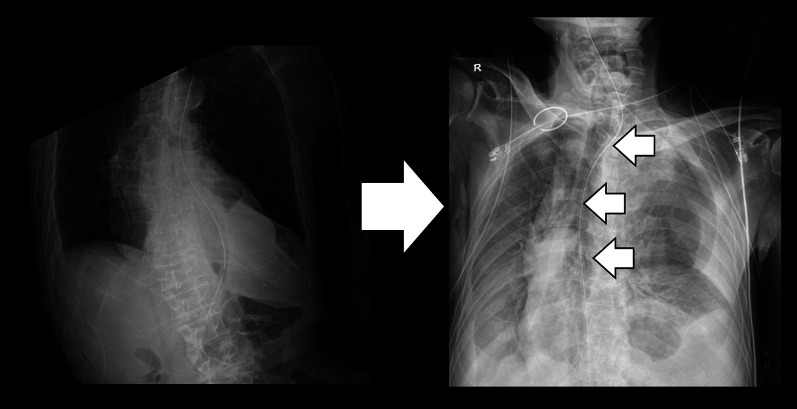
Severe mediastinal shift to the right, suggesting obstructive shock due to intestinal hernia into the thoracic cavity

**Fig. 3:**
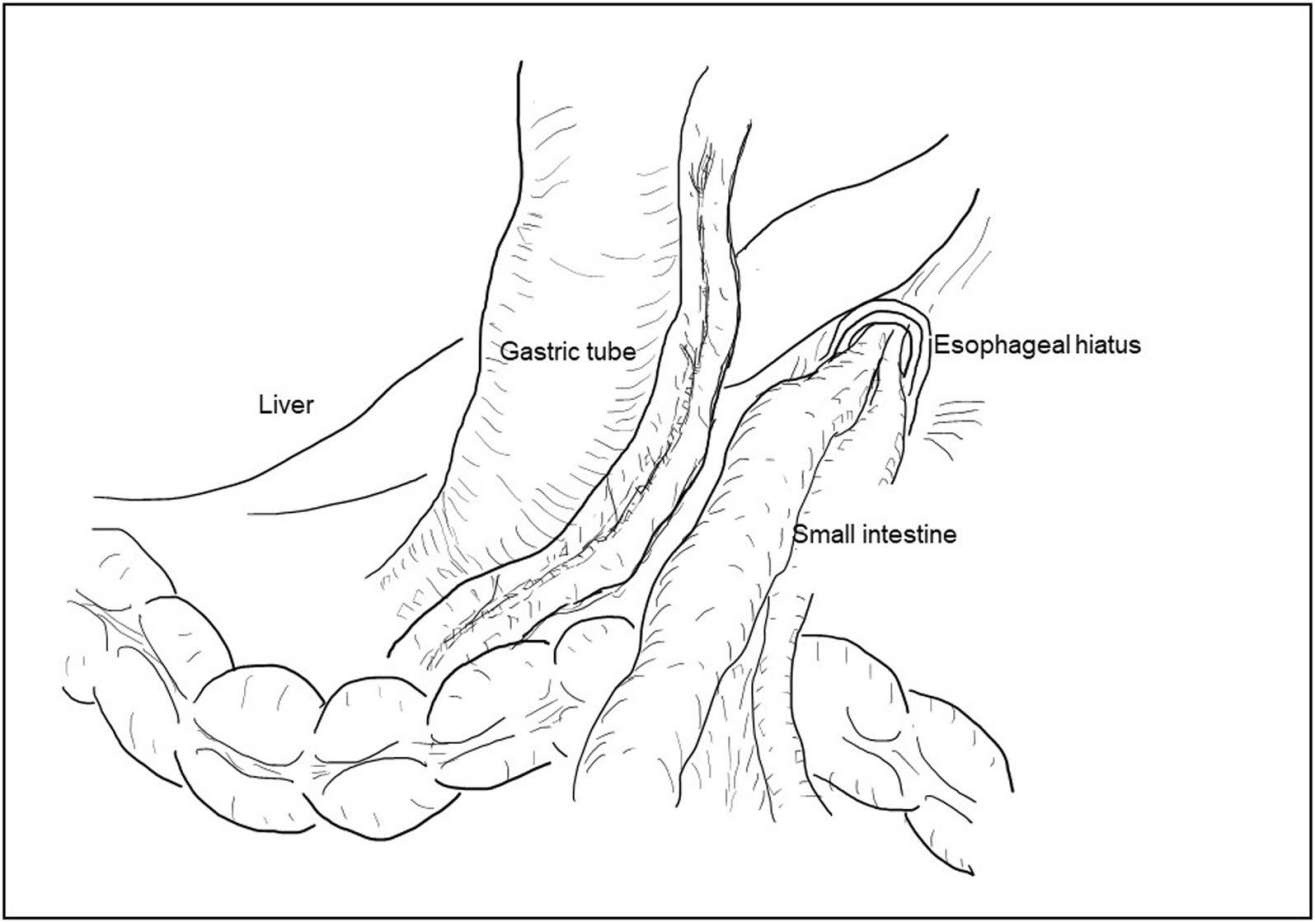
The small intestine had herniated from the esophageal hiatus into the left thoracic cavity, was strangulated by the diaphragmatic crura

An 82-year-old Japanese male (height, 178cm; weight, 56.1kg; BMI, 18.7kg/m^2^) with persistent epigastric pain and nausea was transported to our hospital. He had a history of esophagogastric junction cancer (pT3N0M0 pStageIIA), following neoadjuvant chemotherapy, he had undergone a robotic-assisted thoracoscopic esophagectomy and gastric tube reconstruction via a subcutaneously route with three-field lymphadenectomy at another hospital 3 months prior. He experienced postoperative chylothorax, which improved with conservative management. Following rehabilitation, he was discharged home 7 weeks ago. On arrival at our ER, his oxygen saturation was 98% on room air, pulse rate was 85bpm, blood pressure was 150/80 mmHg. The patient presented with spontaneous pain and tenderness in the upper abdomen. Blood test revealed an elevated white blood cell level of 11110/μL. The remaining blood test results were normal. Computed tomography revealed that the proximal jejunum had herniated through the esophageal hiatus into the left thoracic cavity, with dilation of the subcutaneous gastric tube and duodenum (Fig. 1). He was diagnosed with intestinal obstruction due to diaphragmatic hernia after esophageal surgery. He presented during nighttime hours, was urgently admitted for observation with the intention of elective surgery for the following day or thereafter. A nasogastric tube was inserted. However, nine hours after admission, the patient suddenly complained of difficulty breathing. He subsequently developed shock. His blood pressure was 106/65 mmHg, pulse rate of 150bpm, respiratory rate of 30/min with an O2 saturation of 97% on high-flow nasal cannula FiO2:0.4, cyanosis and peripheral coldness appeared. Arterial blood gas test at the time showed metabolic acidosis, a pH of 7.153, PaCO2 of 30 mmHg, PaO2 of 91 mmHg, BE of −18.3 mmol/L, and lactate level of 10.3 mmol/L. Chest X-ray showed a severe mediastinal shift to the right, suggesting obstructive shock due to intestinal hernia into the thoracic cavity (Fig. 2). It was suggested that not only the respiratory status but also the circulatory dynamics had rapidly worsened, and based on the chest X-ray findings, obstructive shock caused by the herniated small intestine in the thoracic cavity was suspected. Immediate surgical decompression of the thoracic cavity was considered necessary to improve the situation, We planned emergency surgery, but shortly after endotracheal intubation was performed on the ward, he experienced cardiac arrest. The initial waveform indicated ventricular fibrillation (VF), and defibrillation was applied twice. After transitioning to pulseless electrical activity (PEA), resulting in the return of spontaneous circulation (ROSC) 20min later, but continuous adrenaline infusion was required to maintain blood pressure. Surgery was promptly initiated. Here is the surgical schema (Fig. 3). An upper midline abdominal incision was made to avoid the subcutaneous gastric tube. It was observed that the small intestine had herniated from the esophageal hiatus into the left thoracic cavity, where it was strangulated by the diaphragmatic crura. A portion of the diaphragmatic crura was incised to manually reduce the herniated small intestine back into the abdominal cavity. After that, there was some improvement of blood pressure, and as continuous adrenaline infusion could be discontinued, it was diagnosed of obstructive shock due to herniated small intestine. Approximately 2000 mL of bloody pleural fluid was observed in the left thoracic cavity. The herniated small intestine measured approximately 220 cm in length, extending from a point approximately 5 cm distal to the ligament of Treitz. Although the small intestine was congested, the congestion improved and intestinal peristalsis was observed after reduction, leading to the decision not to perform small bowel resection. After aspirating as much bloody pleural fluid as possible, the esophageal hiatus was closed in two layers with 2–0 non-absorbable sutures. Left thoracic and abdominal drainage tubes were placed before closure. The operation time was 102 min. Postoperatively, shock state persisted. Acute kidney injury (AKI) developed, necessitating renal replacement therapy. Additionally, hypoxic-ischemic encephalopathy led to prolonged impaired consciousness. Despite continued intensive care, the patient developed ischemic perforation of the descending colon and aspiration pneumonia, died 29 days after surgery.

## Discussion

Postoperative diaphragmatic hernia can be life-threatening complication. Among the 35 cases, 20 cases (57%) underwent emergency surgery, with 4 cases (20%) resulting in in-hospital mortality, as previously reported [6]. The mortality rate for strangulated diaphragmatic hernias is reported to be 20% [7]. Most likely, many of these cases involve septic shock due to strangulated intestinal obstruction, and numerous cases have been reported. In this case as well, it is considered that there was some degree of ischemia due to strangulation of the small intestine. However, during the acute deterioration, not only did the circulatory dynamics worsen rapidly, but the respiratory status also deteriorated rapidly. This suggests that septic shock due to strangulated intestinal obstruction alone cannot fully explain the clinical condition. The mediastinal deviation observed on chest X-ray suggests that the herniation of the small intestine into the thoracic cavity rapidly progressed from admission to the time of acute deterioration, which is consistent with the worsening respiratory status. Furthermore, the improvement in blood pressure following reduction during surgery suggests that the heart may have been compressed by the herniated intestines. Respiratory dysfunction was caused by lung compression, and increased intrathoracic pressure impaired venous return, resembling a condition similar to tension pneumothorax. Surgical decompression of the thoracic cavity is critical for resolving this condition, making emergency surgery the primary and essential intervention. Consequently, the primary symptom was presumed to be obstructive shock due to rapidly herniation of the intestines. While case reports exist of obstructive shock caused by giant hiatus hernia [8, 9], there are no reports following esophageal surgery.

A diaphragmatic hernia refers to the condition where abdominal or retroperitoneal organs or tissues protrude into the thoracic cavity or mediastinum due to a defect in the diaphragm or enlarged spaces. Postoperative diaphragmatic hernia has been reported following procedures such as hiatal hernia repair, achalasia surgery, and esophageal cancer surgery. It occurs when abdominal organs herniate into the thoracic cavity due to the enlargement of the hiatus during surgery or weakening of the diaphragm due to tumor infiltration, resulting in partial resection [10]. Orr et al. performed a meta-analysis of 6,058 cases and reported symptomatic diaphragmatic hernia rates of 4.5% following thoracoscopic or open esophageal cancer surgery, and 1.0% following open surgery for esophageal cancer [1].

The onset of diaphragmatic hernia is categorized into early (3–8 days postoperatively) and late (6–19 months postoperatively) onset. The causes of early onset cases include surgical manipulation at the hiatal region (such as hiatal enlargement or lateral incision of the diaphragm) [11], increased intra-abdominal pressure due to ileus or enteral nutrition use. Late-onset cases are attributed to the enlargement of the hiatal hernia due to dilation of the gastric tube from pyloric stenosis, increased intra-abdominal pressure from coughing or vomiting. Additionally, regardless of the onset timing, the pressure difference between the thoracic and abdominal cavities has been implicated in the onset of diaphragmatic hernia [12]. It is presumed that pressure differentials are also involved in the onset of this case. Following esophageal surgery, especially in cases where the thoracic and abdominal cavities were contiguous during surgery, it is hypothesized that the rapid herniation of the small intestine into the thoracic cavity is facilitated by the increase in intra-abdominal pressure due to intestinal obstruction and the negative pressure in the thoracic cavity caused by difficulty breathing.

It is suggested that minimally invasive surgeries such as thoracoscopic or laparoscopic approaches may be associated with diaphragmatic hernia following esophageal surgery [6, 13, 14]. The incidence of hiatal hernia requiring surgical intervention following esophagectomy is reported to be 0.73% in open esophagectomy and 1.4% in laparoscopic surgery [15]. As a reason, it has been reported that there is less adhesion between other organs in the abdominal cavity after laparoscopic surgery [12, 16, 17]. With the increasing trend of thoracoscopic surgeries in recent years, if thoracoscopic or laparoscopic surgeries are contributing factors, there may be a possibility of further increase in postoperative diaphragmatic hernias. Especially when performing laparoscopic surgery, it seems necessary to consider hiatal closure, keeping in mind the reduced adhesion. Inquiry at the hospital where the surgery was performed confirmed that, irrespective of whether the procedure was open or thoracoscopic, the esophageal hiatus was sutured in all cases except for reconstructions via the posterior mediastinal route. In this patient’s surgery, the esophageal hiatus was also running sutured using 2–0 monofilament, non-absorbable sutures. During the emergency surgery at our hospital, there was insufficient time to identify the sutures, but a firm, cord-like structure was palpated at the esophageal hiatus, suggesting potential suture rupture. This rupture was likely due to tissue fragility caused by preoperative chemotherapy or advanced age.

## Conclusion

In this case, the rapid progression of small intestine hernia into the thoracic cavity led to obstructive shock. While this is very rare, diaphragmatic hernias following esophageal surgery, as seen in this case, can pose life-threatening risks. Therefore, prompt reduction of strangulated intestine through early surgery is desirable to mitigate such risks.

## Data Availability

Not applicable.
